# CysLT_1_ Receptor Activation Decreases Na^+^/K^+^‐ATPase Activity via PKC‐Mediated Mechanisms in Hippocampal Slices

**DOI:** 10.1111/jnc.70257

**Published:** 2025-10-09

**Authors:** Leonardo Magno Rambo, Quéli Fernandes Lenz, Fernanda Rossatto Temp Fava, Laura Hautrive Milanesi, Joseane Righes Marafiga, Ana Cláudia Jesse, Carlos Fernando Mello

**Affiliations:** ^1^ Programa de Pós‐Graduação em Bioquímica Universidade Federal do Pampa (UNIPAMPA) Uruguaiana Rio Grande do Sul Brazil; ^2^ Departamento de Fisiologia e Farmacologia Programa de Pós‐Graduação em Farmacologia, Centro de Ciências da Saúde, Universidade Federal de Santa Maria (UFSM) Santa Maria Rio Grande do Sul Brazil; ^3^ Department of Neurological Surgery University of California, San Francisco San Francisco California USA

**Keywords:** immunoreactivity, leukotrienes, neurological diseases, PKC, sodium pump, western blot

## Abstract

Leukotrienes (LTs) are potent bioactive lipids derived from the 5‐lipoxygenase (5‐LOX)‐mediated metabolism of arachidonic acid (AA). Growing evidence suggests that leukotrienes contribute to the pathophysiology of several inflammatory disorders of the central nervous system. However, the molecular mechanisms by which cysteinyl leukotrienes (CysLTs) facilitate excitatory activity remain poorly understood. Sodium/potassium‐ATPase (Na^+^/K^+^‐ATPase) is a plasma membrane protein essential for maintaining ionic gradients and regulating membrane excitability, and its reduced activity has been implicated in increased excitability within the central nervous system. In the present study, we demonstrate that LTD_4_ decreases Na^+^/K^+^‐ATPase activity (α_1_ and α_2/3_ subunits) in hippocampal slices from adult male Swiss mice. Furthermore, the intracerebroventricular (i.c.v.) administration of LTD_4_ reduced Na^+^/K^+^‐ATPase activity ex vivo, reinforcing the pathophysiological relevance of our in vitro findings. The LTD_4_‐induced decrease in Na^+^/K^+^‐ATPase activity was prevented by both the CysLT_1_ receptor (CysLT_1_R) inverse agonist montelukast and an anti‐CysLT_1_R antibody, as well as by the PKC inhibitor GF109203X. Moreover, LTD_4_ increased PKC phosphorylation and enhanced Ser‐16 phosphorylation of Na^+^/K^+^‐ATPase. These effects were also prevented by PKC inhibition. In summary, our findings demonstrate that CysLT_1_R activation inhibits hippocampal Na^+^/K^+^‐ATPase activity in mice through a PKC‐dependent mechanism, providing a potential molecular basis for LTD_4_ involvement in the pathophysiology of various neurological disorders.

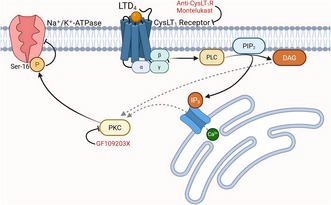

## Graphical abstract

Schematic representation of the molecular mechanism by which leukotriene D_4_ (LTD_4_) inhibits Na^+^/K^+^‐ATPase activity in the hippocampus. LTD_4_ binds to cysteinyl leukotriene receptor 1 (CysLT_1_R), a Gq protein‐coupled receptor, triggering the PLC/PIP_2_ signaling cascade. This leads to the production of diacylglycerol (DAG) and inositol trisphosphate (IP_3_), which promote Ca^2+^ mobilization from the endoplasmic reticulum. DAG and Ca^2+^ jointly activate protein kinase C (PKC), which phosphorylates the Na^+^/K^+^‐ATPase α subunit at Ser‐16, resulting in decreased enzymatic activity. This inhibitory pathway is prevented by CysLT_1_R antagonists (montelukast and anti‐CysLT_1_R antibody) and by the PKC inhibitor GF109203X.

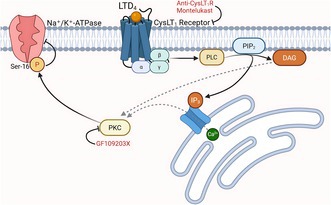

**Abbreviations**
5‐HPETE: 5‐hydroperoxyeicosatetraenoic acid
5‐LOX: 5‐lipoxygenase
AA: arachidonic acid
ANOVA: analysis of variance
BCA: bicinchoninic acid
BSA: bovine serum albumin
CNS: central nervous system
CysLT: cysteinyl leukotriene
CysLT_1_R: cysteinyl leukotriene receptor type 1
CysLT_2_R: cysteinyl leukotriene receptor type 2
DAG: diacylglycerol
DMSO: dimethyl sulfoxide
ECL: enhanced chemiluminescence
EEG: electroencephalogram
GF109203X: bisindolylmaleimide I, a selective PKC inhibitor
i.c.v.: intracerebroventricular
IP_3_
: inositol 1,4,5‐trisphosphate
IP_3_R: inositol 1,4,5‐trisphosphate receptor
LT: leukotriene
LTD_4_
: leukotriene D_4_
PBS: phosphate‐buffered saline
PGE_2_
: prostaglandin E_2_
Pi: inorganic phosphate
PIP_2_
: phosphatidylinositol 4,5‐bisphosphate
PKA: protein kinase A
PKC: protein kinase C
PLC: phospholipase C
PTZ: pentylenetetrazole
RRID: research resource identifier (see scicrunch.org)
SDS‐PAGE: sodium dodecyl sulfate–polyacrylamide gel electrophoresis
SEM: standard error of the mean
SNK: Student–Newman–Keuls
TBS‐T: tris‐buffered saline containing 0.05% Tween 20

## 1 Introduction

Leukotrienes are potent bioactive lipids derived from arachidonic acid (AA) metabolism, synthesized via the 5‐lipoxygenase (5‐LOX) pathway in various tissues, including the mammalian brain, under both physiological and pathological conditions (Tassoni et al. 2008). While leukotrienes are well known for their role in asthma and inflammation, accumulating evidence over the past few years has also linked them to increased neuronal excitability and the pathophysiology of several neurological disorders (Ghosh et al. 2016; Sood et al. 2024). In fact, clinical and experimental studies report elevated cysteinyl leukotrienes in neurological conditions, including traumatic brain injury (Farias et al. 2009), seizures (Simmet and Tippler 1990), multiple sclerosis (Pietrantonio et al. 2024), and meningitis (Matsuo et al. 1996). Inhibiting the leukotriene pathway alleviates neuropathic pain (Zhou et al. 2014), ischemia (Khan et al. 2021; Zhang et al. 2013), neurodegenerative diseases (Michael et al. 2021; Wang et al. 2020), and seizures (Bano et al. 2024; Rehni and Singh 2011). In this context, our group has shown that pranlukast and montelukast reduce PTZ‐induced seizures, while LTD_4_ facilitates them (Lenz et al. 2014), reinforcing the role of cysteinyl leukotrienes in excitotoxicity‐related neurodegeneration. However, the underlying molecular mechanisms involved in this process remain unclear.

Na^+^/K^+^‐ATPase is an integral plasma membrane protein widely expressed in virtually all excitable tissues, including the brain, where it plays a crucial role in Na^+^ electrochemical gradient generation necessary for neurotransmitter uptake from the synaptic cleft and intracellular Ca^2+^ extrusion (Aperia 2012; Horisberger 2004; Lingrel et al. 2003). Thus, a decrease in its activity has been implicated in increased excitability in the central nervous system (Lees 1991). It is well established that Na^+^/K^+^‐ATPase activity is modulated by post‐translational modifications, particularly phosphorylation, induced by several signaling pathways, including PKA and PKC (Poulsen et al. 2010). In this regard, our group has previously demonstrated that prostaglandin E_2_ (PGE_2_), another important eicosanoid, decreases Na^+^/K^+^‐ATPase activity in rat hippocampal slices possibly by Na^+^/K^+^‐ATPase phosphorylation at Ser‐943, a site regulated by PKA; such an effect is reversed by PKA or PKC inhibitors (Oliveira et al. 2009).

CysLT_1_ receptors are Gq protein‐coupled receptors in which PKC serves as the primary downstream kinase effector. Supporting this concept, Foley (1997) has demonstrated that 5‐hydroperoxyeicosatetraenoic acid (5‐HPETE), the main hydroperoxide derivative of arachidonic acid produced by 5‐LOX and a precursor of LTD_4_, inhibits Na^+^/K^+^‐ATPase activity in synaptosomes from the rat cerebral cortex. These findings suggest that 5‐LOX products may regulate neuronal Na^+^/K^+^‐ATPase activity in vivo. Conversely, Sloniewsky et al. (2004) reported that LTD_4_ increases Na^+^/K^+^‐ATPase activity in rat alveolar epithelium, while Satoh et al. (1993) found no effect of LTD_4_ on Na^+^/K^+^‐ATPase activity in the rat cortical collecting duct. These discrepancies highlight the need for further investigation into the effects of LTD_4_ on Na^+^/K^+^‐ATPase activity in different tissues.

Despite these findings, little is known about the effects of cysteinyl leukotrienes and CysLT_1_ receptor activation on cerebral Na^+^/K^+^‐ATPase activity or the underlying molecular mechanisms. Here, we hypothesize that LTD_4_ inhibits Na^+^/K^+^‐ATPase activity in mice hippocampal slices by binding to Gq protein–coupled CysLT_1_ receptors, leading to PKC activation and subsequent Na^+^/K^+^‐ATPase phosphorylation. Therefore, this study aimed to investigate the role of CysLT_1_ receptors in modulating Na^+^/K^+^‐ATPase activity in mouse hippocampal slices and to elucidate the potential mechanisms involved in this process.

## 2 Methods

### 2.1 Reagents

Montelukast (a CysLT_1_R inverse agonist, cat. no. 35779) and LTD_4_ (CysLTRs agonist, cat. no. 20310) were purchased from Cayman Chemical (Ann Arbor, MI, USA). Montelukast was dissolved in 100% dimethyl sulfoxide (DMSO) and then diluted in apyrogenic sterile saline in such a way that the DMSO concentration did not exceed 0.5%. LTD_4_, supplied as an ethanol solution, was evaporated and diluted in artificial cerebrospinal fluid (aCSF). Antibodies were purchased from Santa Cruz Biotechnology (Inc., Santa Cruz, CA, USA) and Abcam (Waltham, MA, USA). The anti‐CysLT1R antibody used for functional blocking was cat. no. sc‐31172, RRID:AB_2088865, from Santa Cruz Biotechnology. PKC inhibitor GF‐109203X (Bisindolylmaleimide I, cat. no. 203290) was purchased from Sigma‐Aldrich (St. Louis, MO, USA). All other reagents were of analytical grade and purchased from local suppliers.

### 2.2 Animals and Sample Size Determination

Adult male Swiss mice (2 months old, 35 ± 3.5 g) (RRID:IMSR_TAC:swi) were obtained from the institutional animal facility (Federal University of Santa Maria, Santa Maria, Brazil). The animals were housed in groups of five per standard polycarbonate cage (30 × 20 × 13 cm) with wood shavings as bedding and kept under standard conditions of temperature and humidity (12 h light/dark cycle, 24°C ± 1°C, 55% relative humidity) with free access to food and water. The experimental protocol was approved by the Institutional Animal Ethics Committee and conducted in accordance with the policies of the National Institutes of Health Guide for the care and use of laboratory animals (#69/2010). No pre‐established exclusion criteria were applied, and no animals were excluded or died during the course of the experiments. All animals completed the planned experimental protocols. In total, 69 mice were used across all experiments. These included animals used for hippocampal slice preparations (*n* = 53), homogenate assays (*n* = 4), and ex vivo i.c.v. experiments (*n* = 12). All efforts were made to minimize the number of animals and their suffering, in accordance with ARRIVE guidelines.

Sample sizes were determined based on pilot data from previously published studies in our laboratory (Oliveira et al. 2009; Rambo et al. 2012). The observed effect of the treatment on Na^+^/K^+^‐ATPase activity was substantial (mean difference: 23.6 units, Cohen's *d* = 2.53). A post hoc power analysis (*α* = 0.05, power = 0.8) indicated that a minimum sample size of *n* = 3 per group would be sufficient to detect an effect of this magnitude. Our final group sizes (*n* = 4–7) were chosen to significantly exceed this minimum requirement. This approach ensures robust statistical power, accounts for potential experimental variability, and aligns with both field standards and the ethical principle of minimizing animal use.

### 2.3 Hippocampal Slices Preparation

Horizontal hippocampal slices were prepared as described by Upreti et al. (2012) with minimal modifications. Mice were euthanized by cervical dislocation, decapitated, and their brains were quickly removed and hemisected in oxygenated ice‐cold (4°C, 95% O_2_/ 5% CO_2_) sucrose‐based cutting solution containing (in mM) 87 NaCl, 25 NaHCO_3_, 25 glucose, 75 sucrose, 2.5 KCl, 1.25 NaH_2_PO_4_, 0.5 CaCl_2_, and 7 MgCl_2_. Horizontal brain slices were cut (380 μm thick) with a vibratome (Leica VT1000S, cat. no. 1491200S001), and hippocampus was gently dissected from each slice. Hippocampal slices were incubated in oxygenated cutting solution at 32°C for 30 min, then transferred to artificial cerebrospinal fluid (aCSF) containing (in mM) 126 NaCl, 3 KCl, 1.25 NaH_2_PO_4_, 1.3 MgCl, 2.5 CaCl_2_, 26 NaHCO_3_, 10 glucose, and maintained at room temperature under constant oxygenation for an additional 60 min to allow stabilization. It is important to note that each brain yielded a sufficient number of hippocampal slices to allocate 6–8 slices per assay tube, allowing one complete set of experimental groups—typically four—from a single animal. Accordingly, all experimental procedures corresponding to one animal (i.e., one subject per group) were performed per day.

### 2.4 Experimental Design and Sample Preparation for Na^+^/K^+^‐ATPase Activity Assay

All experimental procedures were conducted under blinded conditions. The experimenter responsible for performing the pharmacological treatments and ex vivo preparations was not involved in data acquisition or analysis. Enzymatic activity assays and western blot quantification were performed by investigators who were unaware of the treatment allocation. Group codes were revealed only after statistical analyses were completed.

For hippocampal slice and homogenate experiments, each animal served as its own source of tissue and contributed samples to all experimental conditions within a given protocol; therefore, no randomization procedure was required. For ex vivo experiments involving i.c.v. surgery, animals were assigned to receive either LTD_4_ or aCSF by simple alternation during the experimental schedule, ensuring balanced group sizes.

After a 60‐min stabilization period of hippocampal slices, four independent in vitro experimental conditions were investigated:

(1) The effect of CysLT1R agonist, LTD_4_, on intact hippocampal Na^+^/K^+^‐ATPase activity: Hippocampal slices were transferred to a glass test tube containing oxygenated aCSF at 37°C. The effect of CysLT1R agonist on hippocampal Na^+^/K^+^‐ATPase activity was investigated by incubating 6–8 intact hippocampal slices for 30 min at 37°C with increasing concentrations of LTD_4_ (0, 1, 10, or 100 nM). After the incubation period, the medium was discarded, and slices were gently homogenized (10–12 strokes) in ice‐cold 10 mM Tris–HCl buffer (pH 7.4), and Na^+^/K^+^‐ATPase activity was measured.

(2) The effect of LTD_4_ on Na^+^/K^+^‐ATPase activity of hippocampal homogenates: To determine whether LTD_4_ decreases Na^+^/K^+^‐ATPase activity by directly interacting with the enzyme, hippocampal slices were homogenized in ice‐cold 10 mM Tris–HCl buffer (pH 7.4), and LTD_4_ (0, 1, 10, or 100 nM) was added directly to the homogenate‐containing medium.

(3) The effect of montelukast, a CysLT_1_ receptor inverse agonist, on Na^+^/K^+^‐ATPase activity: In those experiments designed to investigate the effect of montelukast on Na^+^/K^+^‐ATPase activity, intact hippocampal slices were incubated with increasing montelukast concentrations (0, 1, 10, or 100 μM) for 30 min at 37°C. In a separate set of this assay, we investigated the effect of montelukast (1 μM) on LTD_4_‐induced decrease in Na^+^/K^+^‐ATPase activity. For this purpose, intact hippocampal slices were preincubated for 15 min with montelukast before LTD_4_ (10 nM). In addition, to present another evidence that LTD_4_ is binding at the CysLT_1_ receptor, we preincubated hippocampal slices for 15 min with the boiled (inactivated) or intact anti‐CysLT_1_R antibody (SC‐31172; 1:1000) before LTD_4_ (10 nM).

(4) The effect of PKC inhibitor, GF‐109203X, on LTD_4_‐induced decrease in Na^+^/K^+^‐ATPase activity: Since the CysLT_1_ receptor is a G_q_‐protein coupled receptor, we investigated the involvement of its main downstream effector, protein kinase C (PKC). First, a concentration‐response curve was performed using the PKC inhibitor GF‐109203X (0.03, 0.3, or 3 μM) to assess its effect on Na^+^/K^+^‐ATPase activity in mouse intact hippocampal slices for 30 min at 37°C. Subsequently, to evaluate the effect of the PKC inhibitor on the LTD_4_‐induced decrease in Na^+^/K^+^‐ATPase activity, intact hippocampal slices were preincubated with PKC inhibitor (GF‐109203X, 0.3 μM) for 15 min prior to LTD_4_ incubation (10 nM).

### 2.5 Na^+^/K^+^‐ATPase Activity Measurements

Na^+^/K^+^‐ATPase activity was measured according to Oliveira et al. (2009). Briefly, the assay medium consisted of (in mM) 30 Tris–HCl buffer, pH 7.4; 0.1 EDTA; 50 NaCl; 5 KCl; 6 MgCl_2_; and 50 μg of protein, in a final volume of 250 μL in the presence or absence of ouabain (3 μM, which inhibits Na^+^/K^+^‐ATPase isoforms containing α_2_ and α_3_ subunits; or 4 mM, which inhibits all isoforms). The reaction was started by the addition of adenosine triphosphate to a final concentration of 4 mM. After 30 min at 37°C, the reaction was stopped by the addition of (*w*/*v*) 50% trichloroacetic acid. Saturating substrate concentrations were used, and the reaction was linear with protein (1 mg/mL) and time (30 min). Appropriate controls were included in the assays for non‐enzymatic hydrolysis of ATP. Inorganic phosphate (Pi) release was quantified colorimetrically, as described by Fiske and Subbarow (1925), with KH_2_PO_4_ as a reference standard. Specific Na^+^/K^+^‐ATPase activity was calculated by subtracting the ouabain‐insensitive activity from the overall activity (in the absence of ouabain) and expressed in nmol Pi/mg protein/min. Alfa_1_‐ Na^+^/K^+^‐ATPase isoform activity was calculated by subtracting the Na^+^/K^+^‐ATPase activity attributed to α_2_ and α_3_ isoforms (3 μM ouabain‐insensitive activity) from the 4 mM ouabain‐sensitive activity (all isoforms).

### 2.6 Ex Vivo Experiments

To determine whether the LTD_4_‐induced decrease of Na^+^/K^+^‐ATPase activity also occurs in vivo, mice were anesthetized with ketamine/xylazine (50/5 mg/kg, i.p.) and a cannula was inserted bilaterally into the lateral ventricles (coordinates relative to bregma: L: +0.9, V: −1.6) (Paxinos and Franklin 2001) with a stereotaxic device. The cannulas were fixed to the skull with dental acrylic cement. Ceftriaxone (100 mg/kg, i.p.) and meloxicam (2 mg/kg, i.p.) were administered immediately before the surgical procedure to prevent infection and reduce perioperative pain. Following cannula implantation, animals were individually monitored at least once daily for seven days to assess recovery, including grooming, locomotor activity, posture, and feeding behavior. Particular attention was given to signs of pain or distress such as reduced mobility, piloerection, or weight loss. No additional analgesic doses were administered after surgery, since meloxicam provided perioperative analgesia and further treatments could interfere with neuroinflammatory and excitability‐related outcomes under investigation. All animals recovered uneventfully, showing normal feeding and locomotor activity within 48 h after surgery, and no signs of infection or severe distress were observed during the monitoring period. Seven days following the surgical procedure, animals were injected with LTD_4_ (2 pmol/μL, i.c.v.). After 30 min, mice were euthanized by decapitation and their brains were quickly removed. The hippocampus was dissected and homogenized to measure Na^+^/K^+^‐ATPase activity. The dose of LTD_4_ was chosen based on Lenz et al. (2014), who demonstrated that i.c.v. administration of LTD_4_ at 0.2–2 pmol reverses the anticonvulsant action of montelukast and modulates seizure threshold and EEG activity, whereas higher doses (6 and 20 pmol) are primarily associated with blood–brain barrier disruption and direct seizure induction. Therefore, 2 pmol was selected as a representative and physiologically relevant dose within the range shown to modulate excitability, while avoiding nonspecific effects on barrier integrity.

### 2.7 Western Blot Assay

Hippocampal slices were incubated under different conditions, as previously described, to evaluate Na^+^/K^+^‐ATPase phosphorylation at Ser16 and p‐Ser16/total Na^+^/K^+^‐ATPase ratio; and PKC/PKA phosphorylation. After the incubation period, the medium was discarded, and slices were homogenized in ice‐cold (4°C) extraction buffer containing (in mM) 25 Tris (pH 7.4), 150 NaCl, and 1% NP‐40, 5% glycerol, 1 NaF, 10 β‐glycerophosphate, 1 DTT, and 2 sodium orthovanadate, supplemented with a protease inhibitors cocktail (Sigma‐Aldrich [St. Louis, MO, USA] cat. no. P8340). The homogenates were centrifuged (12 000 x *g*) for 20 min at 4°C and the supernatants were collected. Protein concentration was determined by the bicinchoninic acid (BCA) method and an aliquot (20 μg protein) of the supernatant was mixed with SDS loading buffer and boiled for 5 min. Proteins were resolved by a 12.5% SDS‐polyacrylamide gel electrophoresis (SDS‐PAGE) and electroblotted using Trans‐Blot Turbo Transfer System (BioRad; RRID: SCR_023156) onto nitrocellulose membranes (Thermo Fisher Scientific, Waltham, MA; cat. no. 88018). A molecular weight standard for calibrating western blots was used (Bio‐Rad; cat. no. 1610375). Membranes were blocked with 5% bovine serum albumin (BSA) plus 5% non‐fat milk in Tris‐buffered saline containing 0.05% Tween 20 (TBS‐T) at room temperature for 1 h (antibodies for phosphorylated protein were blocked only with BSA). After specific blocking, membranes were washed with TBS‐T twice at room temperature for 10 min and then incubated overnight at 4°C with the following primary antibodies diluted in TBS‐T plus BSA (5%) and/or non‐fat milk (5%), depending on the primary antibody (antibodies for phosphorylated protein diluted only with 5% BSA): anti‐Na^+^/K^+^‐ATPase α subunit (1:1000; Santa Cruz Biotechnology, cat. no. sc‐21 712, RRID: AB_626713); anti‐phospho‐Na^+^/K^+^‐ATPase α1 (Ser16) (1:1000; Abcam, cat. no. ab194532); anti‐PKC α (C‐20) (1:150000; Santa Cruz Biotechnology, cat. no. SC‐208; RRID: AB_2168668); anti‐phospho‐PKC α (Ser657) (1:30000; Santa Crus Biotechnology, cat. no. SC‐12356; RRID: AB_2168557); anti‐PKA Iα reg (C‐14) (1:10000; Santa Cruz Biotechnology, cat. no. SC‐18800; RRID: AB_2284174); anti‐phospho‐PKA IIα reg (Ser96) (1:10000; Santa Cruz Biotechnology, cat. no. SC‐12905; RRID: AB_2237413). Anti‐β‐actin (C4) (1:100000; Santa Cruz Biotechnology, cat. no. SC‐47778; RRID: AB_626632) and anti‐GAPDH (1:1000; Santa Cruz Biotechnology, cat. no. sc‐32 233; RRID: AB_627679) served as a loading control. After primary antibody incubation, membranes were washed twice with TBS‐T at room temperature for 15 min. This procedure was followed by incubation with goat anti‐rabbit IgG‐horseradish peroxidase conjugated secondary antibody (1:3000; Santa Cruz Biotechnology, cat. no. SC‐2004; RRID: AB_631746) diluted in TBS‐T containing 5% BSA at room temperature for 1 h, followed by three additional washes in TBS‐T at room temperature for 10 min. Blots were developed by enhanced chemiluminescence (ECL) (Thermo Fisher Scientific, Waltham, MA, cat. no. 32106) and the specific band optical densitometry was quantified by ImageJ (version 1.47v or 1.51n; National Institutes of Health [NIH], Bethesda, MD, RRID: SCR_003070). The resultant data were normalized for the control group optical densitometry values and expressed as a relative amount. Membranes were reprobed after stripping with 0.5 M NaCl in 0.2% SDS/TBS at 60°C for 50 min. For experiments in which both phosphorylated and non‐phosphorylated proteins were analyzed, membranes were sequentially stripped and reprobed with the corresponding antibodies. In these cases, the same β‐actin band from the original gel was used for normalization, since it served as the common loading control for all sequential detections on that membrane. Consequently, in the representative blots shown in Figure 4, the β‐actin panels are identical, reflecting the use of the same lanes and normalization control across the different probings.

### 2.8 Protein Determination

The protein content of the samples was determined using the bicinchoninic acid (BCA) method (Thermo Fisher Scientific Inc., Rockford, IL, USA, cat. no. 23225) using BSA as a standard.

### 2.9 Statistical Analysis

The normality of data distribution was assessed using the Shapiro–Wilk test, and homoscedasticity was verified with the Brown–Forsythe test to ensure that all assumptions for ANOVA were met. All datasets satisfied the assumption of normality (*p* > 0.05). No formal test for outliers was conducted, and no data points were excluded from the analyses. Therefore, data were analyzed by one‐ or two‐way ANOVA, or Student's t‐test (ex vivo experiments) according to experimental design, followed by Student–Newman–Keuls (SNK) post hoc test using GraphPad Prism software version 6.07 (San Diego, CA, USA; RRID:SCR_002798). A probability of *p* < 0.05 was considered significant. All data are expressed as mean + S.E.M.

## 3 Results

**FIGURE 1:**
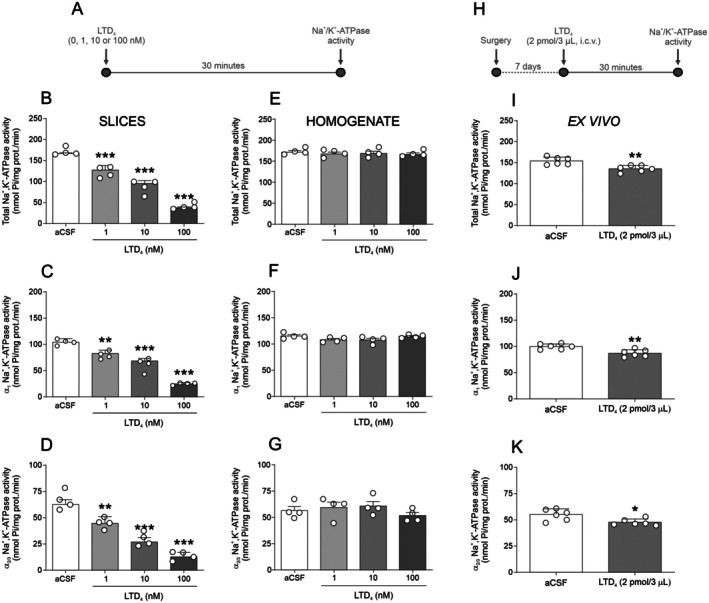
Effect of leukotriene D_4_ (LTD_4_) on Na^+^/K^+^‐ATPase activity in hippocampal slices, homogenate and in ex vivo preparations. (A) Timeline of the experimental protocol used for panels B–G. (B–D) LTD_4_ concentration‐response curve on Total (B), α_1_ (C), and α_2/3_ (D) Na^+^/K^+^‐ATPase activity in hippocampal slices (*n* = 4 animals; hippocampal slices from 4 independent animals; see Methods for details of slice allocation per condition). (E–G) LTD_4_ concentration‐response curve on Total (E), α_1_ (F), and α_2/3_ (G) Na^+^/K^+^‐ATPase activity in hippocampal homogenate (*n* = 4 animals). (H) Timeline of the ex vivo experimental protocol used for panels I–K. Intracerebroventricular (i.c.v.) administration of LTD_4_ on: (I–K) Total (I), α_1_ (J), and α_2/3_ (K) Na^+^/K^+^‐ATPase activity in ex vivo hippocampal preparations (*n* = 6 animals per group). Data are expressed as mean ± standard error of the mean (SEM). *p < 0.05, **p < 0.01, ***p < 0.001 versus artificial cerebrospinal fluid (aCSF) (one‐way analysis of variance [ANOVA] followed by Student–Newman–Keuls [SNK] post hoc test for B–G; unpaired Student's *t*‐test for I–K).

To evaluate the effect of CysLT1 receptor activation on Na^+^/K^+^‐ATPase activity, we first performed a concentration‐response curve for LTD_4_, the primary CysLT1R agonist. We employed a classical pharmacological approach that differentiates Na^+^/K^+^‐ATPase isoforms based on their sensitivity to ouabain: 3 μM inhibits α2/3, while 4 mM inhibits all isoforms (Nishi et al. 1999). Figure 1 illustrates the effects of increasing LTD_4_ concentrations (0, 1, 10, and 100 nM) on Na^+^/K^+^‐ATPase activity. Figure 1A illustrates the timeline used in the experimental design for panels 1B‐G, while Figure 1H presents the timeline for the ex vivo experiments shown in panels 1I‐K. Statistical analysis revealed that incubation of hippocampal slices with LTD_4_ (at all tested concentrations) for 30 min significantly decreased total [F(3,12) = 84.00; *p* < 0.0001; Figure 1B], α_1_ [F(3,12) = 88.50; *p* < 0.0001; Figure 1C], and α_2/3_ [F(3,12) = 49.17; *p* < 0.0001; Figure 1D] Na^+^/K^+^‐ATPase activity.

To confirm that LTD_4_‐induced decrease in Na^+^/K^+^‐ATPase activity resulted from a direct interaction with the enzyme or required intact cellular signaling, we incubated hippocampal homogenates with LTD_4_ (0, 1, 10, or 100 nM) for 30 min. Under these conditions, LTD_4_ did not decrease Na^+^/K^+^‐ATPase activity (total [F(3,12) = 0.2925; *p* = 0.8301; Figure 1E], α_1_ [F(3,12) = 2.598; *p* = 0.1006; Figure 1F], α_2/3_ [F(3,12) = 0.9270; *p* = 0.4575; Figure 1G]). This suggests that the LTD_4_‐induced decrease in Na^+^/K^+^‐ATPase activity is not due to a direct interaction with the enzyme but likely involves a functional signaling cascade.

To assess whether this effect also occurs in vivo, mice were injected i.c.v. with LTD_4_ (2 ρmol/μL), a dose that does not affect behavior per se (Lenz et al. 2014). 30 min post‐injection, Na^+^/K^+^‐ATPase activity was measured in the hippocampus. Statistical analysis confirmed that i.c.v. LTD_4_ administration reduced total [t(10) = 3.844; *p* = 0.0032; Figure 1I; *n* = 6], α_1_ [t(10) = 3.668; *p* = 0.0043; Figure 1J], and α_2/3_ [t(10) = 2.880; *p* = 0.0164; Figure 1K] Na^+^/K^+^‐ATPase activity, reinforcing the biological relevance of the in vitro findings.

**FIGURE 2:**
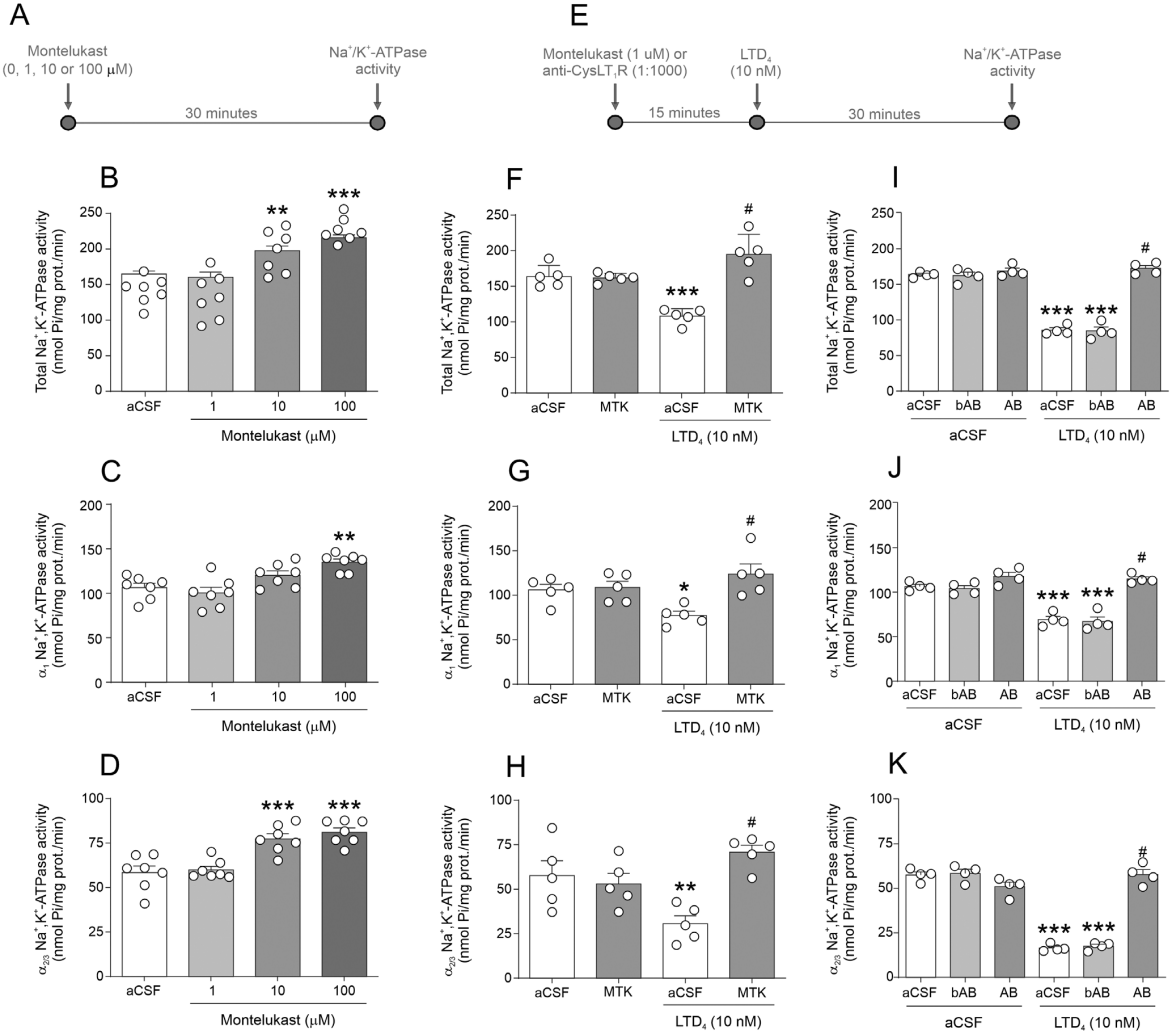
Effect of Montelukast or anti‐CysLT_1_ receptor (cysteinyl leukotriene receptor type 1) on LTD_4_ (leukotriene D_4_)‐induced inhibition of Na^+^/K^+^‐ATPase activity in hippocampal slices. (A) Timeline of the experimental protocol for panels B–D. (B–D) Montelukast concentration‐response curve on Total (B), α_1_ (C), and α_2_/_3_ (D) Na^+^/K^+^‐ATPase activity (*n* = 7 animals; hippocampal slices from 7 independent animals; see Methods for details of slice allocation per condition) (E) Timeline of the experimental protocol for panels F–K. (F–H) Effect of Montelukast pre‐incubation on LTD_4_‐induced Na^+^/K^+^‐ATPase inhibition: Total (F), α_1_ (G), and α_2_/_3_ (H) Na^+^/K^+^‐ATPase activity (*n* = 5 animals). (I–K) Effect of anti‐CysLT_1_ antibody pre‐incubation on LTD_4_‐induced Na^+^/K^+^‐ATPase inhibition: Total (I), α_1_ (J), and α_2_/_3_ (K) Na^+^/K^+^‐ATPase activity (*n* = 4 animals). Data are expressed as mean ± standard error of the mean (SEM). *p < 0.05, **p < 0.01, ***p < 0.001 versus respective artificial cerebrospinal fluid (aCSF); #*p* < 0.05 versus LTD_4_ + aCSF (one‐way analysis of variance [ANOVA] followed by Student–Newman–Keuls [SNK] post hoc test for B–D; two‐way ANOVA followed by SNK's post hoc test for F–K).

To further investigate the involvement of CysLT1 in LTD_4_ effect on Na^+^/K^+^‐ATPase activity, we performed a concentration‐response analysis for montelukast, a CysLT1 inverse agonist. Figure 2 shows the effects of three different concentrations of montelukast (0, 1, 10, and 100 μM) on Na^+^/K^+^‐ATPase activity in hippocampal slices. Figure 2A depicts the timeline used in the experimental design for Figure 2B–D, while Figure 2E shows the timeline corresponding to the experiments presented in Figure 2F–K. Incubation with montelukast for 30 min increased α_2/3_ Na^+^/K^+^‐ATPase activity at 10 μM [F(3,28) = 7.97; *p* = 0.0007; Figure 2D], while at 100 μM it increased total [F(3,28) = 7.81; *p* = 0.0006; Figure 2B] and α_1_ [F(3,28) = 10.73; *p* < 0.0001; Figure 2C] activity. For the reversal experiments, we selected the concentration without a significant effect on Na^+^/K^+^‐ATPase activity (1 μM).

Pre‐incubation of slices with montelukast (1 μM) for 15 min prior to LTD_4_ (10 nM) prevented the LTD_4_‐induced decrease in Na^+^/K^+^‐ATPase activity (total [F(1,16) = 32.85; *p* < 0.0001; Figure 2F], α_1_ [F(1,16) = 8.127; *p* = 0.0116; Figure 2G] and α_2/3_ [F(1,16) = 14.52; *p* = 0.0015; Figure 2H]). Additionally, pre‐incubation with an intact anti‐CysLT1R antibody (1:1000) prevented the LTD_4_‐induced decrease in total Na^+^/K^+^‐ATPase activity [F(2,18) = 81.98; *p* < 0.0001; Figure 2I], α_1_ [F(2,18) = 38.35; *p* < 0.0001; Figure 2J] and α_2/3_ [F(2,18) = 59.66; *p* < 0.0001; Figure 2K], whereas the boiled antibody had no effect. Altogether, these findings suggest that LTD_4_ mediates the inhibition of Na^+^/K^+^‐ATPase activity through CysLT1 activation.

**FIGURE 3:**
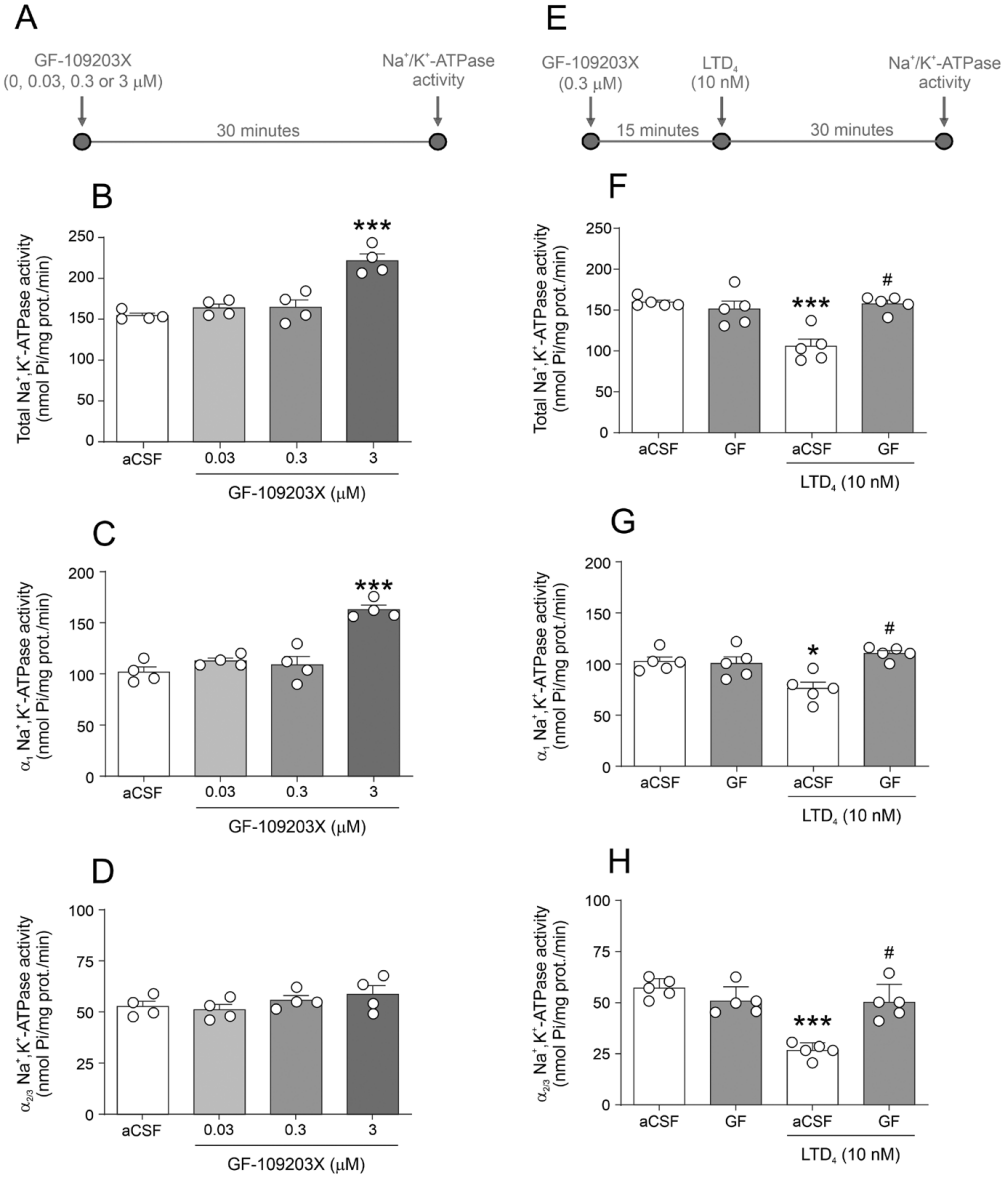
Effect of PKC (protein kinase C) inhibition on LTD_4_ (leukotriene D_4_)‐induced Na^+^/K^+^‐ATPase activity inhibition in hippocampal slices. (A) Experimental timeline for panels B–D. (B–D) GF‐109203X (bisindolylmaleimide I, a selective PKC inhibitor) concentration‐response curve on Total (B), α_1_ (C), and α_2_/_3_ (D) Na^+^/K^+^‐ATPase activity (*n* = 4 animals; hippocampal slices from 4 independent animals; see Methods for details of slice allocation per condition). (E) Timeline for panels F–H. (F–H) Effect of GF‐109203X pre‐incubation on LTD_4_‐induced Na^+^/K^+^‐ATPase inhibition: Total (F), α_1_ (G), and α_2_/_3_ (H) Na^+^/K^+^‐ATPase activity (*n* = 5 animals). Data are expressed as mean ± standard error of the mean (SEM). *p < 0.05, ***p < 0.001 versus respective artificial cerebrospinal fluid (aCSF); #*p* < 0.05 versus LTD_4_ + aCSF (one‐way analysis of variance [ANOVA] followed by Student–Newman–Keuls [SNK] post hoc test for B–D; two‐way ANOVA followed by SNK post hoc test for F–H).

Next, we investigated whether downstream signaling pathways of CysLT1R are involved in LTD_4_‐induced inhibition in Na^+^/K^+^‐ATPase activity. Given that PKC is a key downstream kinase in CysLT1R signaling and a critical modulator of Na^+^/K^+^‐ATPase activity (Poulsen et al. 2010), we examined the effect of a PKC inhibitor on LTD_4_‐mediated inhibition of Na^+^/K^+^‐ATPase activity. Figure 3A presents the timeline used in the experimental design for Figure 3B–D, while Figure 3E shows the timeline corresponding to the experiments shown in Figure 3F–H. Figure 3B–D present a concentration‐response curve for the PKC inhibitor GF‐109203X (0, 0.03, 0.3, and 3 μM). Statistical analysis showed that only the highest concentration (3 μM) significantly increased total [F(3,12) = 20.52; *p* < 0.0001; Figure 3B] and α_1_ [F(3,12) = 25.53; *p* < 0.0001; Figure 3C] activity, but not α_2/3_ [F(3,12) = 1.218; *p* = 0.3455; Figure 3D]. We therefore selected 0.3 μM, the highest concentration without intrinsic effects, for the reversal experiments. Pre‐incubation with GF‐109203X (0.3 μM) 15 min before LTD_4_ (10 nM) prevented LTD_4_‐induced decrease in total [F(1,24) = 29.9; *p* < 0.0001; Figure 3F] and α_1_ [F(1,24) = 49.35; *p* < 0.0001; Figure 3G] activity, but not α_2/3_ [F(1,24) = 4.20; *p* = 0.9949; Figure 3H], suggesting that PKC mediates the LTD_4_‐induced effects on Na^+^/K^+^‐ATPase activity.

**FIGURE 4:**
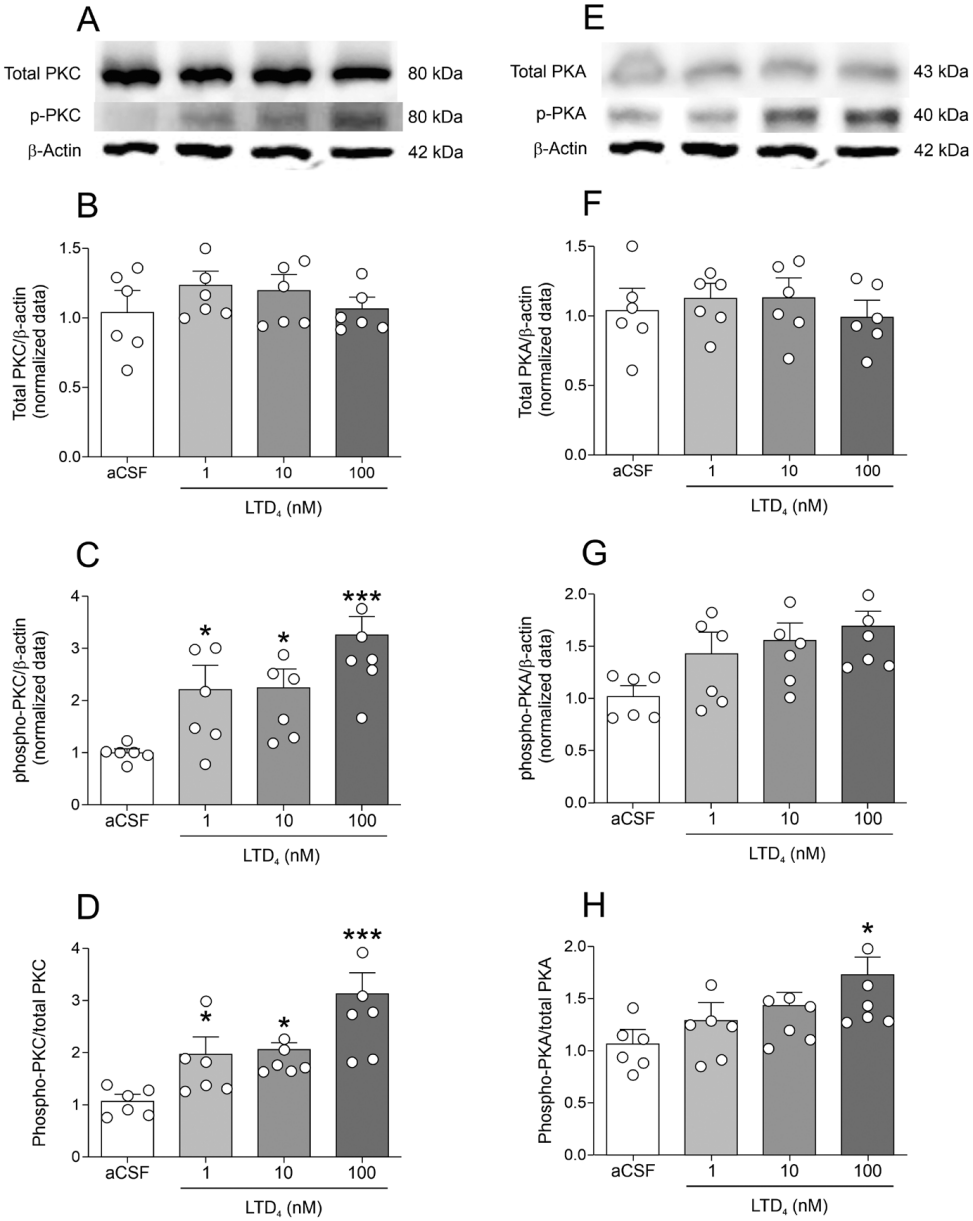
Effect of LTD_4_ (leukotriene D_4_) on PKC (protein kinase C) and PKA (protein kinase A) phosphorylation in hippocampal slices. (A) Representative western blot images for the analyses in panels B–C. (B–D) LTD_4_ concentration‐response curve on total PKC immunoreactivity (B), phospho‐PKC immunoreactivity (C), and the phospho‐PKC/total PKC ratio (D) (*n* = 6 animals; hippocampal slices from 6 independent animals; see Methods for details of slice allocation per condition). (E) Representative western blot images for panels F–G. (F–H) LTD_4_ concentration‐response curve on total PKA immunoreactivity (F), phospho‐PKA immunoreactivity (G), and the phospho‐PKA/total PKA ratio (H) (*n* = 6 animals). β‐Actin in panels A and E corresponds to the same membrane since these samples were resolved in the same gel and probed simultaneously. Data are expressed as mean ± standard error of the mean (SEM). *p < 0.05, ***p < 0.001 versus artificial cerebrospinal fluid (aCSF) (one‐way analysis of variance [ANOVA] followed by Student–Newman–Keuls (SNK) post hoc test).

To further explore signaling pathways involved in LTD_4_ effects, we examined PKC and PKA phosphorylation in hippocampal slices incubated with LTD_4_ (0, 1, 10, or 100 nM). Figure 4A shows representative western blot images for Figure 4B–C, and Figure 4E shows representative bands for Figure 4F–G. LTD_4_ significantly increased phosphorylated PKCα (Ser657) at all concentrations tested [F(3,20) = 7.083; *p* = 0.0020; Figure 4C] and phosphorylated PKA IIα regulatory subunit (Ser96) only at 100 nM [F(3,20) = 3.301; *p* = 0.0414; Figure 4G], without affecting total PKC [F(3,20) = 0.648; *p* = 0.5931; Figure 4B] or total PKA [F(3,20) = 0.257; *p* = 0.8549; Figure 4F]. Consequently, phospho‐PKC/total‐PKC [F(3,20) = 9.284; *p* = 0.0005; Figure 4D] increased at all concentrations, while phospho‐PKA/total‐PKA [F(3,20) = 3.275; *p* = 0.0424; Figure 4H] increased only at 100 nM.

**FIGURE 5:**
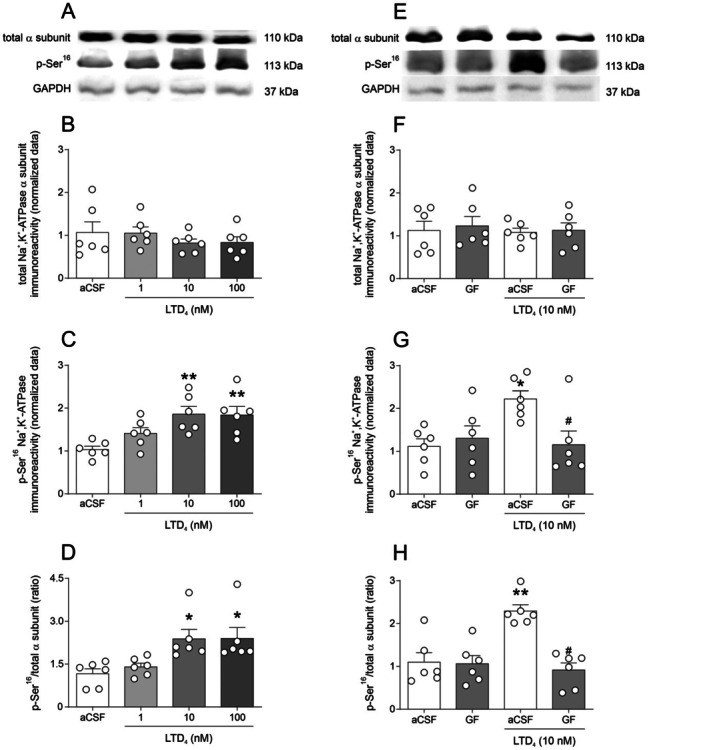
Effect of PKC (protein kinase C) inhibition on LTD_4_ (leukotriene D_4_)‐induced Na^+^/K^+^‐ATPase phosphorylation at Ser^16^ in hippocampal slices. (A) representative western blot images for panels B–C. (B–D) LTD_4_ concentration‐response curve on total Na^+^/K^+^‐ATPase α subunit immunoreactivity (B), p‐Ser^16^ Na^+^/K^+^‐ATPase immunoreactivity (C), and p‐Ser^16^/total α subunit ratio (D) (*n* = 6 animals; hippocampal slices from 6 independent animals; see Methods for details of slice allocation per condition). (E) representative western blot images for panels F, G. (F, G) Effect of GF‐109203X (bisindolylmaleimide I, a selective PKC inhibitor) pre‐incubation in LTD_4_ effects on total Na^+^/K^+^‐ATPase α subunit immunoreactivity (F), p‐Ser^16^ Na^+^/K^+^‐ATPase immunoreactivity (G) and in the p‐Ser^16^/total α subunit ratio (H) (*n* = 6 animals). Data are expressed as mean ± standard error of the mean (SEM). *p < 0.05, **p < 0.01 versus artificial cerebrospinal fluid (aCSF); #*p* < 0.05 versus LTD_4_ + aCSF (one‐way analysis of variance (ANOVA) followed by SNK post hoc test for B–D; two‐way ANOVA followed by Student–Newman–Keuls (SNK) post hoc test for F–H).

Lastly, we assessed whether LTD_4_ modulates PKC‐mediated phosphorylation of Na^+^/K^+^‐ATPase, which could result in decreased enzymatic activity. Figure 5A shows representative western blot images for Figure 5B,C, and Figure 5E shows representative bands for Figure 5F,G. Figure 5 shows that LTD_4_ (10 and 100 nM) increases Na^+^/K^+^‐ATPase phosphorylation at Ser^16^ [F(3,20) = 6.436; *p* = 0.0032; Figure 5C] and p‐Ser^16^/total α subunit Na^+^/K^+^‐ATPase ratio [F(3,20) = 5.522; *p* = 0.0063; Figure 5D], but did not alter total Na^+^/K^+^‐ATPase immunoreactivity [F(3,20) = 0.658; *p* = 0.5872; Figure 5B]. Pre‐incubation with the PKC inhibitor GF‐109203X prevented the LTD_4_‐induced increase in p‐Ser^16^ immunoreactivity [F(1,20) = 4.779; *p* = 0.0409; Figure 5G] and restored the p‐Ser^16^/total α subunit ratio to control levels [F(1,20) = 13.50; *p* = 0.0015; Figure 5H], without affecting total α subunit expression [F(1,20) = 0.0249; *p* = 0.8760; Figure 5F]. These findings indicate that LTD_4_ enhances Ser^16^ phosphorylation of Na^+^/K^+^‐ATPase via a PKC‐dependent pathway, without altering total protein levels. Therefore, our data support that LTD_4_ induces PKC‐mediated phosphorylation, which in turn leads to Na^+^/K^+^‐ATPase phosphorylation and a subsequent decrease in its activity.

## 4 Discussion

In the present study, we demonstrated that incubation with LTD_4_ decreases Na^+^/K^+^‐ATPase activity in mouse hippocampal slices in an isoform‐independent manner. Notably, this inhibitory effect was absent in hippocampal homogenates, suggesting that an intact cellular environment is necessary for the downstream signaling cascade. This hypothesis was further supported by our in vivo experiments, in which i.c.v. administration of LTD_4_ reproduced the inhibitory effect on Na^+^/K^+^‐ATPase activity observed in hippocampal slices, underscoring the biological relevance of our findings. Additionally, we identified the CysLT_1_ receptor as a key mediator of this effect, as both the inverse agonist montelukast and an anti‐CysLT_1_R antibody prevented the LTD_4_‐induced inhibition of Na^+^/K^+^‐ATPase activity, implicating this receptor and its downstream signaling pathway in this response. Given that PKC is the main effector of the CysLT_1_ receptor signaling, we explored the role of PKC in this process and found that PKC inhibition with GF‐109203X abolished the LTD_4_‐induced decrease in Na^+^/K^+^‐ATPase activity. Furthermore, LTD_4_ exposure increased the p‐PKC/PKC ratio and enhanced phosphorylation at Ser‐16 of Na^+^/K^+^‐ATPase, an effect that was also prevented by PKC inhibition. Interestingly, activation of PKA was observed only at the highest concentration of LTD_4_ (100 nM), suggesting that Na^+^/K^+^‐ATPase activity may also be modulated secondarily by PKA activation in this context. Indeed, we cannot discard an interplay between PKA and PKC on Na^+^/K^+^‐ATPase phosphorylation (Cheng et al. 1997).

The reduction of Na^+^/K^+^‐ATPase activity by LTD_4_ is consistent with previous studies indicating that inflammatory mediators can modulate ion transporters and contribute to neuronal hyperexcitability. Given the crucial role of Na^+^/K^+^‐ATPase in maintaining ion homeostasis and neuronal excitability (McGrail et al. 1991), its inhibition by LTD_4_ may create an imbalance in electrochemical gradients, promoting excitatory neurotransmission and increasing susceptibility to pathological conditions such as epilepsy (Sun et al. 2022). Our findings align with growing evidence implicating CysLT_1_ receptors in various neurological conditions, including ischemia (Zhang et al. 2013), epilepsy (Rehni and Singh 2011), and neurodegenerative diseases such as Parkinson's disease (Wang et al. 2020; Wallin and Svenningsson 2021). For instance, Ding et al. (2007) demonstrated that CysLT_1_ receptor expression is upregulated in neurons following brain cryoinjury, with the pharmacological antagonism of this receptor mitigating injury‐related damage. Similarly, montelukast has been shown to protect against Na^+^/K^+^‐ATPase inhibition and oxidative stress in models of acute pancreatitis (Ozkan et al. 2010), reinforcing the idea that CysLT_1_ receptor blockade can preserve Na^+^/K^+^‐ATPase function under pathological conditions.

The potential involvement of CysLT_2_ receptors in the effects of LTD_4_ on Na^+^/K^+^‐ATPase activity cannot be fully overlooked. Evidence suggests that activation of CysLT_2_ receptors by LTD_4_ may be involved in various neurological disorders (Ghosh et al. 2016). The CysLT_2_ receptors blockade with a specific antagonist, such as HAMI 3379, can confer neuroprotection in models of focal cerebral ischemia (Zhang et al. 2013), as well as the blockade using a dual antagonist of CysLT_1_R and CysLT_2_R, BAYu9773, may prevent BBB disruption and seizures (Lenz et al. 2014). Notably, the anticonvulsant effects of BAY‐u9773 appear to be partial, as it did not affect latency for myoclonic jerks, unlike those of selective CysLT_1_R antagonists, thus supporting a primary role for CysLT_1_R in modulating excitability. Although montelukast and the anti‐CysLT_1_R antibody strongly support a CysLT_1_‐mediated mechanism, we cannot entirely rule out the involvement of CysLT_2_ receptors, given that LTD_4_ has some affinity for this receptor. In our experiments, LTD_4_‐induced Na^+^/K^+^‐ATPase inhibition and phosphorylation were completely abolished by both montelukast and an anti‐CysLT_1_R antibody, strongly supporting CysLT_1_R as the receptor mediating these effects. If CysLT_2_R were involved, one would expect some degree of residual phosphorylation or enzymatic inhibition after CysLT_1_ blockade, which was not observed. Thus, although definitive exclusion of CysLT_2_R would require further studies with selective antagonists (e.g., HAMI 3379) or genetic knockdown strategies, our findings strongly indicate that CysLT_1_R is the primary receptor mediating LTD_4_‐induced modulation of Na^+^/K^+^‐ATPase activity. These enzymatic changes may have downstream functional consequences on neuronal excitability.

The modulation of Na^+^/K^+^‐ATPase by arachidonic acid (AA) or its metabolites has been previously described in other tissues. Singh et al. (2012) reported that AA inhibits Na^+^/K^+^‐ATPase activity in the pulmonary vasculature via PKC‐dependent mechanisms. Our results provide additional evidence for PKC involvement in the regulation of Na^+^/K^+^‐ATPase activity in the hippocampus, with CysLT_1_ receptor activation leading to PKC‐dependent inhibition of the enzyme. Furthermore, different AA derivatives exert distinct effects on Na^+^/K^+^‐ATPase activity depending on the pathway involved (Foley 1997; Koide et al. 1986), suggesting a complex interplay between lipid mediators and ion transport regulation in the brain.

Although our findings provide compelling evidence that LTD_4_ decreases hippocampal Na^+^/K^+^‐ATPase activity via CysLT_1_ receptor activation and PKC‐dependent signaling, some limitations should be considered. First, our experiments were conducted exclusively in male mice, which precludes any inference regarding potential sex‐dependent differences in leukotriene signaling or Na^+^/K^+^‐ATPase regulation. Future studies should include both sexes to assess possible sex‐specific responses. Second, while we demonstrated that LTD_4_ does not affect Na^+^/K^+^‐ATPase activity in homogenates, further studies employing selective inhibitors of intracellular signaling intermediates other than PKC (e.g., PLC, IP_3_R, or PKA) could help map the upstream signaling cascade. In particular, because CysLT_1_R is known to couple to the canonical Gq/PLC/IP_3_/Ca^2+^ pathway, it will be important to confirm whether LTD_4_‐induced Na^+^/K^+^‐ATPase inhibition involves PLC activation and intracellular Ca^2+^ mobilization. Approaches such as Ca^2+^ imaging with Fluo‐4 or Fura‐2 AM, combined with pharmacological inhibition of PLC (U73122) or IP_3_R (xestospongin C), will be essential to directly test this possibility in future studies. Furthermore, the observed activation of PKA at high concentrations of LTD_4_ raises questions about possible off‐target or concentration‐dependent effects not fully explored in this study. Lastly, although our study focused on biochemical mechanisms, it is important to consider the functional implications of LTD_4_ signaling in the CNS. In this regard, Lenz et al. (2014) demonstrated that LTD_4_ (2 pmol, i.c.v.) does not modify EEG amplitude by itself, but critically reversed the anticonvulsant effect of montelukast in a PTZ‐induced seizure model. This finding indicates that CysLT_1_R activation by LTD_4_ can directly influence neuronal excitability. Taken together, these data support the notion that the PKC‐dependent inhibition of hippocampal Na^+^/K^+^‐ATPase that we describe here may represent a mechanistic substrate underlying the excitability changes observed at the behavioral and electrophysiological levels. Future studies will be directed at testing this hypothesis directly through electrophysiological recordings in hippocampal slices.

Taken together, our results suggest that LTD_4_, via CysLT_1_ receptor activation, decreases hippocampal Na^+^/K^+^‐ATPase activity through a PKC‐dependent phosphorylation at the Ser‐16 residue of the α catalytic subunit. This inhibitory effect was observed both in vitro and ex vivo and was abolished by pharmacological blockade of the CysLT_1_ receptor and PKC. The lack of effect in hippocampal homogenates underscores the necessity of intact intracellular signaling. This effect may contribute to increased neuronal excitability associated with inflammatory conditions, offering a potential therapeutic target for neurological disorders in which inflammation‐derived 5‐LOX products play a role. In fact, our results suggest that targeting the leukotriene pathway could represent a potential therapeutic strategy for neurological conditions such as epilepsy, neuroinflammation, and neurodegeneration.

## Author Contributions

**Leonardo Magno Rambo:** conceptualization, investigation, writing – original draft, writing – review and editing, methodology, validation, formal analysis, data curation, supervision, project administration. **Quéli Fernandes Lenz:** conceptualization, methodology, validation, investigation, formal analysis. **Fernanda Rossatto Temp Fava:** investigation, validation, methodology. **Laura Hautrive Milanesi:** investigation, validation, methodology. **Joseane Righes Marafiga:** investigation, validation, methodology, writing – review and editing. **Ana Cláudia Jesse:** investigation, validation, methodology. **Carlos Fernando Mello:** conceptualization, funding acquisition, writing – original draft, writing – review and editing, supervision, project administration.

## Conflicts of Interest

The authors declare no conflicts of interest.

## Data Availability

The datasets generated during and/or analyzed during the current study are available from the corresponding author on reasonable request.
